# Oral Vaccination of White-Tailed Deer (*Odocoileus virginianus*) with *Mycobacterium bovis* Bacillus Calmette-Guerin (BCG)

**DOI:** 10.1371/journal.pone.0097031

**Published:** 2014-05-07

**Authors:** Mitchell V. Palmer, Tyler C. Thacker, W. Ray Waters, Suelee Robbe-Austerman

**Affiliations:** 1 National Animal Disease Center, Agricultural Research Service, United States Department of Agriculture, Ames, Iowa, United States of America; 2 National Veterinary Services Laboratories, Animal and Plant Health Inspection Service, United States Department of Agriculture, Ames, Iowa, United States of America; University of Minnesota, United States of America

## Abstract

Wildlife reservoirs of *Mycobacterium bovis* represent serious obstacles to the eradication of tuberculosis from livestock, particularly cattle. In Michigan, USA tuberculous white-tailed deer transmit *M. bovis* to other deer and cattle. One approach in dealing with this wildlife reservoir is to vaccinate deer, thus interfering with the intraspecies and interspecies transmission cycles. Thirty-three white-tailed deer were assigned to one of two groups; oral vaccination with 1×10^8^ colony-forming units of *M. bovis* BCG Danish (n = 17); and non-vaccinated (n = 16). One hundred eleven days after vaccination deer were infected intratonsilarly with 300 colony-forming units of virulent *M. bovis*. At examination, 150 days after challenge, BCG vaccinated deer had fewer gross and microscopic lesions, fewer tissues from which *M. bovis* could be isolated, and fewer late stage granulomas with extensive liquefactive necrosis. Fewer lesions, especially those of a highly necrotic nature should decrease the potential for dissemination of *M. bovis* within the host and transmission to other susceptible hosts.

## Introduction

*Mycobacterium bovis* is the cause of tuberculosis in animals and has a broad host range that includes cattle, deer and humans. Human infection can result in disease indistinguishable from that caused by *M. tuberculosis*. During the early to mid 20^th^ century, public health concerns posed by zoonotic transmission of *M. bovis* from cattle to humans prompted many countries to implement national programs to eradicate tuberculosis from cattle [1]. In most developed nations eradication campaigns have been successful in decreasing the prevalence of bovine tuberculosis. Nevertheless, in spite of long-standing and costly efforts, some countries have found it impossible to eradicate tuberculosis from the cattle population. One obstacle responsible for many faltering eradication campaigns is the presence of a wildlife reservoir of *M. bovis* infection [2]. In most cases, wildlife became infected when *M. bovis* “spilled over” from cattle decades ago during periods of high disease prevalence in cattle; however, in many regions, *M. bovis* is now “spilling back” from wildlife to cattle, impeding the progress of eradication [3], [4]. Although some wildlife species are dead-end hosts, inconsequential in maintenance of the disease within a region and transmission to cattle (i.e. spillover hosts), other species are capable of maintaining disease and transmitting *M. bovis* to other susceptible hosts (i.e. maintenance hosts). Recognized wildlife maintenance hosts of *M. bovis* include the brushtail possum (*Trichosurus vulpecula*) in New Zealand, European badger (*Meles meles*) in the United Kingdom and Republic of Ireland, wild boar (*Sus scrofa*) in Spain, African buffalo (*Syncerus caffer*) in South Africa and white-tailed deer (*Odocoileus virginianus*) in the United States (USA). Control of disease in wildlife maintenance hosts frequently involves efforts to decrease the size and density of wild populations. Often successful in decreasing disease prevalence, such efforts have been unsuccessful in eradication of disease from wildlife or cattle [5]. In an effort to diminish wildlife to cattle transmission of *M. bovis* some countries are investigating the possible role of wildlife vaccination [6]–[13].

In 1994, a hunter-harvested, free-ranging white-tailed deer (*Odocoileus virginianus*) in Michigan (MI), USA was diagnosed with tuberculosis due to *M. bovis* [14]. Subsequent surveys in the region identified a focus of *M. bovis* infection in free-ranging white-tailed deer in northeast MI [15]. This represented the first known reservoir of *M. bovis* in wildlife in the USA, the first time white-tailed deer served as an *M. bovis* maintenance host population and not least of all, a serious impediment to the ongoing effort to eradicate bovine tuberculosis from USA cattle. At least 60 cattle herds in MI have been diagnosed with tuberculosis since the discovery of tuberculosis in free-ranging deer, presumably from direct or indirect contact with infected deer. Typing of DNA shows *M. bovis* isolates from deer and cattle to be the same, suggesting a common source of infection [16], [17]. Surveillance and control measures, including decreasing the MI deer population through increased hunting, have been in place for over 15 years and a significant reduction in apparent prevalence of tuberculosis in deer has been achieved [17]. However, hunter support for further population reduction is waning and public resentment of control measures is growing [17].

A control measure that could be applied to specific areas of sustained high disease prevalence is vaccination of deer to prevent infection, disease, or transmission. In white-tailed deer, parenteral vaccination with *M. bovis* Bacille Calmette Guerin (BCG) Danish and BCG Pasteur has demonstrated decreased disease severity, but not prevention of infection. Boosting an initial dose 6 weeks later did not raise the level of protection [18], [19]. In a pilot study, orally administered BCG Danish reduced lesion severity but did not prevent tissue colonization by *M. bovis* [20]. The purpose of the current study was to expand on the pilot study and evaluate the protective effect of orally administered BCG Danish followed by experimental infection with virulent *M. bovis*.

## Materials and Methods

### Animals, Vaccination and Challenge

Thirty-three white-tailed deer (∼1 yr.-old) were obtained from a captive breeding herd (tuberculosis free) at the National Animal Disease Center (NADC) in Ames, Iowa, USA. Deer were randomly assigned to one of two groups; orally vaccinated with 1×10^8^ colony-forming units (CFU) *M. bovis* BCG Danish (n = 17); and non-vaccinated (n = 16). Deer were vaccinated as previously described [20]. Briefly, with the aid of a swine mouth speculum, a 1.0 ml preparation of 1×10^8^ CFU BCG in phosphate buffered saline (PBS) was administered to the posterior oropharynx using a 3-ml syringe and a 10 French 25-cm sterile urinary catheter (Monoject, St. Louis, MO, USA). All deer were experimentally infected 111 days after vaccination. Infection was intratonsilar, as described previously, with approximately 150 CFU of virulent *M. bovis* strain 1315 (low passage) placed into each palatine tonsilar crypt for a total dose of 300 CFU per deer [21]. Strain 1315 (NADC designation), used for challenge, was originally isolated from a white-tailed deer in MI. For challenge infection, deer were anesthetized by IM injection of a combination of xylazine (2 mg/kg) (Mobay Corporation, Shawnee, KS) and ketamine (6 mg/kg) (Fort Dodge Laboratories, Fort Dodge, IA). After intratonsilar infection the effects of xylazine were reversed by IM injection of tolazoline (4 mg/kg) (Lloyd Laboratories, Shenandoah, IA).

Deer were housed separately, by vaccination status inside a biosafety level 3 (BL-3) building with personnel wearing appropriate personal protective equipment, including full-face respirators with HEPA filtered canisters to prevent exposure to aerosolized *M. bovis*. The BL-3 animal housing had negative air pressure as compared to the outside. Airflow was such that air was pulled out of individual rooms, preventing air exchange between rooms. Flow was adjusted to produce 11.4 air changes per hour. This study was carried out in strict accordance with the Guide for the Care and Use of Laboratory Animal of the National Institutes of Health and the Guide for the Care and Use of Agricultural Animals in Agricultural Research and Teaching of the Federation of Animal Science Societies. The NADC Institutional Animal Care and Use Committee approved protocols prior to implementation.

### Challenge Inoculum and Vaccines

The BCG vaccine as well as the virulent *M. bovis* challenge strain were grown in Middlebrook’s 7H9 media supplemented with 10% oleic acid-albumin-dextrose complex (Difco, Detroit, MI) plus 0.05% Tween 80 (Sigma Chemical Co., St. Louis, MO) as described [22]. Mid log-phase growth bacilli were pelleted by centrifugation at 750×*g*, washed twice in PBS (0.01 M, pH 7.2), and diluted to the appropriate cell density in PBS. Bacilli were enumerated by serial dilution plate counting on Middlebrook’s 7H11 selective media (Becton Dickinson, Cockeysville, MD). A single vaccine dose consisted of 10^8^ CFU *M. bovis* BCG Danish in 1.0 ml PBS, while a single challenge dose consisted of 300 CFU *M. bovis* 1315 in 0.2 ml PBS.

### Necropsy and Tissue Sampling

One hundred fifty days after challenge with virulent *M. bovis* all deer were euthanized by IV sodium pentobarbital. At necropsy, the following tissues were collected and processed for isolation of *M. bovis* and microscopic analysis as described [23], palatine tonsil, lung, liver; mandibular, parotid, medial retropharyngeal, tracheobronchial, mediastinal, hepatic, mesenteric and superficial cervical lymph nodes.

Lymph nodes were cross-sectioned at 0.5 cm intervals and examined. Each lung lobe was examined separately and cross-sectioned at 0.5 to 1.0 cm intervals. Lungs and lymph nodes were subjected to semi-quantitative scoring of gross lesions adapted from Vordermeier *et al* [24]. Lung lobes (left cranial, left caudal, right cranial, right caudal, middle and accessory) were subjected to the following scoring system: (0) no visible lesions; (1) no external gross lesions, but lesions seen upon slicing; (2) <5 gross lesions of <10 mm in diameter; (3) >5 gross lesions of <10 mm in diameter; (4) >1 distinct gross lesion of >10 mm in diameter; (5) coalescing gross lesions. Scoring of lymph node gross lesions was based on the following scoring system: (0) no visible lesions; (1) small focal lesion (1–2 mm in diameter); (2) several small foci; (3) extensive lesions. Tissues collected for microscopic analysis were fixed by immersion in 10% neutral buffered formalin. Formalin-fixed tissues were processed for microscopic examination by routine paraffin-embedment techniques, cut in 5 µm sections and stained with hematoxylin and eosin. Adjacent sections were cut from samples containing tuberculous granulomas and stained by the Ziehl-Neelsen technique for visualization of acid-fast bacteria (AFB). Microscopic lesions were staged (stages I–IV) according to criteria adapted from that described by Rhoades *et al* [25]. Stage I (initial) granulomas were characterized by accumulations of epithelioid macrophages admixed with low numbers of lymphocytes and neutrophils. Multinucleated giant cells may be present but necrosis is absent. When present, AFB were seen within macrophages or multinucleated giant cells. Stage II (solid) granulomas were characterized by accumulations of epithelioid macrophages surrounded by a thin, incomplete connective tissue capsule. Infiltrates of neutrophils and lymphocytes were sometimes present at the granuloma periphery as well as multinucleated giant cells. Necrosis when present was minimal. When present, AFB were seen within macrophages or multinucleated giant cells. Stage III (necrotic) granulomas were characterized by necrotic cores surrounded by a zone of epithelioid macrophages admixed with multinucleated giant cells and lymphocytes, all surrounded by a thin fibrous capsule. When present, AFB were seen within macrophages or multinucleated giant cells as well as within the necrotic core. Stage IV (necrotic and mineralized) granulomas were characterized by a thin fibrous capsule surrounding irregular multicentric granulomas with multiple necrotic cores. Necrotic cores contained foci of dystrophic mineralization. Epithelioid macrophages and multinucleated giant cells surrounded necrotic areas and there were often moderate to marked infiltrates of lymphocytes. Acid-fast bacilli were often present in moderate numbers and primarily located within the caseum of the necrotic core.

### Isolation and Identification of Mycobacterial Isolates

Tissues were processed for mycobacterial isolation as previously described [26] using both the BACTEC 460 radiometric system and BACTEC Mycobacteria Growth Indicator Tube (MGIT) 960 system (Becton Dickinson). Isolates were identified by a combination of Ziehl-Neelsen acid-fast staining, and nucleic acid probes (AccuProbe, Gen-Probe, San Diego, CA), spoligotyping and a differential PCR described previously [27]. Further identification of atypical mycobacteria was done using partial 16 S ribosomal sequencing of the ribosomal polymerase β-subunit as described previously [28], [29]. Sequences were then identified through use of a mycobacterial species sequence database and GenBank [30].

### Statistical Analysis

Mean group values for lesion scores were compared using an unpaired Student’s *t*-test with Welch’s correction (GraphPad Prism, GraphPad Software, San Diego, CA, USA). Fisher’s exact test (GraphPad Prism) was used to compare differences in the presence of non-tuberculous mycobacteria (NTM) in vaccinated deer compared to non-vaccinated deer. A *p*-value<0.05 was considered significant.

## Results

Among vaccinated deer, 2/17 displayed gross lesions, in comparison to 11/16 in the non-vaccinated group (Table 1). Vaccinated deer had lesions only in the medial retropharyngeal lymph nodes, while non-vaccinated deer most commonly had lesions in both tracheobronchial (5) and medial retropharyngeal lymph nodes (4). In non-vaccinated deer, lesions were also seen in the mediastinal lymph nodes (2) and lung (2). Given that vaccinated deer only had gross lesions in the medial retropharyngeal lymph nodes, comparison of lesion scores was only possible for this lymph node. Although medial retropharyngeal lymph node scores were higher in the non-vaccinated group (0.44±0.22) than the vaccinated group (0.12±0.08), this difference was not statistically significant, *p*>0.10.

**Table 1:** Summary of gross, microscopic and bacteriological results of tissues from BCG-vaccinated (V) and non-vaccinated (NV) deer after experimental challenge with virulent *M. bovis*.

Deer	Group	GrossLesion	Location	Microscopiclesions	Location	Culture	Location
260	NV	Neg	NA	Neg	NA	*M. bovis*	mrln, tbln
261	NV	Pos	tbln, lung	Neg	NA	*M. bovis*	tbln, lung
1069	NV	Pos	tbln	Pos	tbln	No isolation	NA
1102	NV	Pos	mrln	Pos	ton, mrln	*M. bovis*	mrln, ton
1103	NV	Neg	NA	Pos	mrln, hepln	*M. bovis*	ton, maln, paln, medln, lung,hepln, scln
1113	NV	Pos	lung	Pos	mrln, lung	*M. bovis*	mrln, tbln, lung, mesln
1115	NV	Pos	medln	Pos	medln	No isolation	NA
1120	NV	Pos	mrln	Pos	mrln	No isolation	NA
1142	NV	Pos	tbln	Pos	tbln	*M. kansasii*	tbln
1143	NV	Neg	NA	Neg	NA	*M. nonchromogenicum*	mrln
1150	NV	Pos	mrln	Pos	mrln	*M. bovis*	mrln
1155	NV	Pos	mrln	Pos	ton, mrln	*M. bovis*	mrln, ton
1160	NV	Pos	medln, tbln,lung	Pos	lung	*M. bovis*	tbln
1170	NV	Pos	tbln	Pos	mrln, tbln	*M. bovis*	mrln
1118	V	Pos	mrln	Pos	mrln, tbln	No Isolation	NA
1125	V	Neg	NA	Neg	NA	*M. kansasii*	tbln
1141	V	Neg	NA	Neg	NA	*M. bovis*	mrln
1144	V	Neg	NA	Neg	NA	*M. kansasii*	tbln
1151	V	Neg	NA	Neg	NA	*M. bovis*	ton, mrln
1152	V	Neg	NA	Pos	mrln	*M. bovis, M. intracellulare*	ton, mrln(*M. intracellulare*- mesln)
1156	V	Neg	NA	Neg	NA	*M. avium hominissuis*	medln
1159	V	Neg	NA	Neg	NA	*M. avium complex*	tbln
1161	V	Pos	mrln	Pos	mrln	*M. bovis*	mrln
1167	V	Neg	NA	Neg	NA	*M. bovis*	ton, mrln
1171	V	Neg	NA	Pos	mrln	No isolation	NA

Deer in which there were no gross or microscopic lesions and no bacteriological isolation results have been excluded.

NA = not applicable; Neg = no lesion or no bacteriological isolation; Pos = lesion present or isolation of mycobacteria; mrln = medial retropharyngeal lymph node; tbln = tracheobronchial lymph node; ton = palatine tonsil, maln = mandibular lymph node; paln = parotid lymph node; medln = mediastinal lymph node, hepln = hepatic lymph node; scln = superficial cervical lymph node; mesln = mesenteric lymph node.

Microscopic lesions compatible with tuberculous granulomas were seen in 4/17 vaccinates and 11/16 non-vaccinates. All 4 vaccinates had microscopic lesions in the medial retropharyngeal lymph nodes. A single vaccinate had additional microscopic lesions in the tracheobronchial lymph node. Among non-vaccinates, microscopic lesions were most common in the medial retropharyngeal (7) and tracheobronchial (3) lymph nodes. Microscopic lesions were also seen in the lung (2) and tonsil (2) with a single non-vaccinate having tuberculous granulomas in the hepatic lymph node. Non-vaccinated deer had a greater number of microscopic lesions in stages II–IV in the medial retropharyngeal lymph nodes than did vaccinated deer (Table 2).

**Table 2:** Total number of microscopic granulomas in representative sections of medial retropharyngeal lymph nodes of BCG-vaccinated and non-vaccinated deer after experimental challenge with virulent *M. bovis*.

	Histologic Stage^1^			
Group	I	II	II	IV
Non-vaccinates	26	9	5	11
Vaccinates	9	0	0	0

1 Stage I (initial) granulomas, Stage II (solid) granulomas, Stage III (necrotic) granulomas, Stage IV (necrotic, mineralized, coalescent) granulomas. Complete definitions found in text.

*Mycobacterium bovis* was isolated from 9/16 non-vaccinated deer in a total of 22 different tissues. The number of infected tissues from individual deer ranged from one to seven. Most common tissues from which *M. bovis* was isolated were medial retropharyngeal (6) and tracheobronchial (4) lymph nodes and lung (3). Isolation of *M. bovis* was also successful from tonsil (3) and mediastinal (1), mesenteric (1), parotid (1), mandibular (1), hepatic (1) and superficial cervical (1) lymph nodes.

In contrast, *M. bovis* was isolated from 5/17 vaccinated deer in a total of 8 individual tissues. *Mycobacterium bovis* was isolated from one or two tissues from individual deer. The most common samples from which *M. bovis* was isolated were medial retropharyngeal lymph nodes (5) and tonsils (3). Non-tuberculous mycobacteria were isolated from 5/17 vaccinated deer and 2/16 non-vaccinated deer. Most commonly *M. kansasii* (3) and *M. avium* complex (3) species were isolated, with a single isolation of *M. nonchromogenicum*. The difference in presence of NTM between vaccinated and non-vaccinated deer was not statistically significant (*p* = 0.43).

In vaccinated deer, intralesional AFB were present in low numbers (<10) per tissue section. Similar numbers of AFB were seen in most lesions from non-vaccinated deer; however, in 3 cases AFB were present in larger numbers; deer 1102 (>10 but <20) and deer 1113 and 1155 (>100) per tissue section examined. In all cases AFB were most commonly seen within the necrotic caseum; many times within foci of dystrophic mineralization.

## Discussion

Vaccines for wildlife have been used, or considered for use, in diseases that negatively affect public health, livestock health/commerce or endangered species [31]. These include rabies in skunks, raccoons, foxes and other mammals [32], Lyme disease in mice [33], plague in black-tailed prairie dogs [34], brucellosis in bison and elk [35], classical swine fever in European wild boar [36], anthrax in cheetah and black rhinoceros [37], and tuberculosis in deer, badgers, brushtail possums and wild boar [7]–[10], [18]. Generally, oral vaccines in the form of baits are the most feasible means of vaccinating wildlife. However, under certain circumstances hand injected or pneumatic dart administered vaccines have also been used successfully [8], [35].

In the context of tuberculosis in deer, BCG is the most widely investigated vaccine, having been tested in several species of the Family Cervidae [7], [18], [38]. In almost all species tested, BCG reduced disease (i.e. lesion) severity but did not provide sterile immunity and protect against infection [18], [19], [39], [40]. Multiple studies in New Zealand red deer demonstrated that a single parenteral dose of BCG Pasteur reduced disease severity but did not protect against infection [7], [41]. Although, protection from both infection and disease was seen using a prime-boost regime with low (10^4^ CFU) to moderate (10^6^ CFU) doses of BCG, administered parenterally, 8 or 16 weeks apart [40]. Parenteral vaccination of white-tailed deer with either BCG Danish or Pasteur resulted in decreased disease severity, without sterile immunity [19]. A booster dose 6 weeks later did not raise the level of protection [18].

A previous pilot study with white-tailed deer showed that oral administration of BCG Danish decreased lesion severity after challenge with virulent *M. bovis* [20]. The pilot study used vaccine and challenge doses similar to those in the present study and the results are comparable. Although, in the pilot study the most common site for lesion development after challenge was the mediastinal lymph nodes; with inconsistent lesions in the medial retropharyngeal lymph nodes. The reason for such a difference in lesion distribution is unclear; however, the challenge strain used in the pilot study was 9839 (NADC designation). Strain 9839 is the result of infection of a calf with strain 1315. The effect of passage of *M. bovis* through another host species is unknown.

Efficacious oral vaccination with BCG has been demonstrated in wildlife such as brushtail possums, Eurasian badgers and wild boar [10]–[12], [42], and also in humans. In fact, from 1921 to the 1950s, oral vaccination of human infants during the first 10 days of life was the preferred method of administration across Europe, Asia, Canada and South America [43]. Vaccination of neonatal cattle with BCG provides superior protection to that seen with vaccination at 6 months of age [44]. It is unknown if oral vaccination of neonatal deer affords similar enhanced protection. The wild nature of the targeted deer, as well as the environment, makes neonatal vaccination of deer improbable.

The mode of oral vaccine delivery represents a significant challenge to wildlife vaccination efforts. The ideal vaccine delivery device maintains vaccine viability, directs vaccination toward the target population and not other animals that may have unintended contact with the vaccine (i.e. non-target species) and results in vaccination of a large percentage of the target population (i.e. adequate coverage). Potential oral BCG delivery devices for wildlife include vaccine contained within a lipid formulation [20], [45], a matrix of feed, paraffin and attractant (cinnamon-truffle powder) [46], [47] and a molasses-based bait [48]. To date, none of the delivery devices tested in deer meet the qualifications for a successful vaccine delivery method.

In the present study NTM were isolated from 5/17 vaccinated deer and 2/16 non-vaccinated deer. Non-tuberculous mycobacteria are isolated from deer with some frequency, with members of the *M. avium* complex being the most commonly isolated NTM from deer [45], [49]. Mycobacteria other than those of the *M. avium* or *M. tuberculosis* complexes have also been isolated [50]. In most instances, these isolations have not been associated with lesions; although, *M. kansasii* has caused lesions in deer similar to those induced by *M. bovis* [51]. All three isolations of *M. kansasii* in the current study were from tracheobronchial lymph nodes. Of the three, *M. kansasii* was associated with a tuberculosis-like lesion in one case. Although NTM have been reported in BCG vaccinated and non-vaccinated white-tailed deer in previous vaccine efficacy studies, a greater presence of NTM in BCG vaccinated deer, as in the present study, has not been noted [18]–[20]. In the present study lack of additional data and a greater number of animals precludes any definitive conclusion, leaving one to assume the observed difference is random.

It is not clear what effect colonization with *M. avium* complex or NTM has on vaccine-induced immune responses or responses to the pathogen. In calves, it is believed that exposure to NTM has a negative effect on vaccine-induced protection as cross-reactive responses clear BCG before a protective immune response is mounted [44]. This is unlikely in the present study as more NTM isolations were obtained from the vaccinated group where greater protection was observed; moreover in one case, NTM and *M. bovis* were isolated from the same animal. Thus from this and other studies [7], [19], it appears that colonization with NTM does not negatively affect responses to BCG vaccination in white-tailed deer.

Tuberculous granulomas in deer have less fibrous encapsulation and more extensive necrosis than granulomas from cattle [23], [52]. Indeed, lesions in deer often resemble abscesses, where liquefactive necrosis dominates over the typical caseous necrosis seen in tuberculous granulomas of cattle [23]. Lesions in deer generally contain more AFB than those of cattle [52]. It has also been demonstrated that advanced granulomas (Stages III and IV) with more extensive necrosis, are more likely to contain large numbers of AFB [18]. In the present study, similar to previous studies in white-tailed deer, vaccination with BCG resulted in fewer advanced granulomas [18], [19]. It is plausible that advanced granulomas characterized by liquefied contents, limited fibrous encapsulation and large numbers of AFB are more likely to result in dissemination within the host and excretion with transmission to other susceptible hosts. A vaccine that decreases or prevents the formation of such lesions would likely result in decreased intraspecies and interspecies transmission of *M. bovis*. It follows, that a tuberculosis vaccine for wild deer need not provide sterile immunity to be effective in decreasing disease prevalence through decreased transmission.
